# CCNB2 is a novel prognostic factor and a potential therapeutic target in low-grade glioma

**DOI:** 10.1042/BSR20211939

**Published:** 2022-01-28

**Authors:** Dengfeng Wang, Hongjiao Sun, Xiaohui Li, Gang Wang, Guizhong Yan, Haijun Ren, Boru Hou

**Affiliations:** 1 Department of Neurosurgery, Lanzhou University Second Hospital, Lanzhou, Gansu 730030, P.R. China; 2 Key Lab of Neurology of Gansu Province, Lanzhou, Gansu 730030, P.R. China; 3 VIP Clinic, Lanzhou University Second Hospital, Lanzhou, Gansu 730030, P.R. China

**Keywords:** bioinformatics, CCNB2, diagnosis and prognosis biomarkers, immune infiltrates, Low-grade glioma

## Abstract

**Background:** Cyclin B2 (CCNB2) is an important component of the cyclin pathway and plays a key role in the occurrence and development of cancer. However, the correlation between prognosis of low-grade glioma (LGG), CCNB2, and tumor infiltrating lymphocytes is not clear. **Methods:** The expression of CCNB2 in LGG was queried in Gene Expression Profiling Interactive Analysis 2 (GEPIA2) and TIMER databases. The relationships between CCNB2 and the clinicopathological features of LGG were analyzed using the Chinese Glioma Genome Atlas (CGGA) database. The relationship between CCNB2 expression and overall survival (OS) was evaluated by GEPIA2. The correlation between CCNB2 and LGG immune infiltration was analyzed by the TIMER database. Finally, quantitative real-time polymerase chain reaction (qRT-PCR) was performed to detect CCNB2 expression. **Results:** The expression of CCNB2 differed across different tumor tissues, but was higher in LGG than in normal tissues. LGG patients with high expression of CCNB2 have poorer prognosis. The expression of CCNB2 was correlated with age, WHO grade, IDH mutational status, 1p/19q codeletion status, and other clinicopathological features. The expression of CCNB2 in LGG was positively correlated with the infiltration level of B cells, dendritic cells, and macrophages. qRT-PCR results revealed that the expression of CCNB2 in LGG tissues was higher than normal tissues and higher expression of CCNB2 was associated with worse prognosis. **Conclusion:** CCNB2 may be used as a potential biomarker to determine the prognosis of LGG and is also related to immune infiltration.

## Introduction

Low-grade glioma (LGG) is a common primary malignant tumor, which accounts for ∼20% of primary brain tumors [1]. Clinically, surgical resection, chemotherapy, radiotherapy, and other methods are mainly used for LGG, but inevitably, drug resista nce and recurrence affect the final prognosis of patients [2]. Some patients may rapidly develop into high-grade glioblastoma (GBM) [4]. Thus, it is urgent to find a new biomarker with high accuracy able to predict the prognosis of LGG.

Cyclin (CCN) family members are the key regulators of cell cycle progression and play a regulatory role in different stages of the cell cycle. Their abnormal expression often leads to tumorigenesis. For example, the abnormal expression of CCND1 and CCNE1 promotes the proliferation of lung adenocarcinoma, the down-regulation of cyclin A2 affects the proliferation of colon cancer cells [6]; however, cyclin A2 is highly expressed in thyroid cancer [7]. This evidence reflects the diversity of cyclin A2 effects. Cyclin E1 is considered a potential target for the treatment of ovarian cancer [8]. These studies indicate that cyclins play an important role in the progression of various cancers.

Cyclin B2 (CCNB2), a member of the cyclin family, mainly plays a role in G_2_/M transformation and is up-regulated in human cancer [9,10]. For example, the overexpression of CCNB2 is associated with poor prognosis of HCC [11], while the decreased expression of CCNB2 inhibits the invasion and metastasis of bladder cancer [12]. Ni et al. showed that CCNB2 may act as a biomarker and potential target of lung adenocarcinoma (LUAD) [13]. These results suggest that CCNB2 may be a new strategy for the treatment of cancer; however, the specific mechanism by which CCNB2 regulates the occurrence and development of LGG is still unknown.

In the present study, we evaluated the differential expression of CCNB2 mRNA in LGG tissues and normal tissues, and used the Human Protein Atlas (HPA) to verify the expression of CCNB2 in different tissue types and cell lines. The relationship between the expression of CCNB2 and the prognosis of patients with LGG was analyzed using CCGA and Gene Expression Profiling Interactive Analysis 2 (GEPIA2) databases. In addition, since the immune microenvironment plays a key role in the development of LGG [14,15], we also mined the potential correlation between CCNB2 and the level of immune infiltration using the TIMER database, detected the biological process involved in CCNB2 by gene enrichment analysis and studied the mechanisms of CCNB2 in LGG. Finally, we verified the expression of CCNB2 in LGG tissues and normal tissues by RT-qPCR, and divided patients with LGG into high- and low-expression groups to evaluate the relationships between CCNB2 expression and prognosis of patients.

## Methods

### Tissue samples

We collected 40 LGG cancerous and paracancerous tissue samples together with associated clinical information from the medical records at Lanzhou Second Hospital. None of the subjects underwent radiotherapy or chemotherapy prior to the surgery. All patient materials were obtained with informed consent, and the present study was carried out with the approval of the Clinical Research Ethics Committee.

### Quantitative real-time polymerase chain reaction

TRIzol reagent (Invitrogen, Carlsbad, CA, U.S.A.) was used to extract total RNA from tissues, which was reverse transcribed into cDNA using a RevertAid First Strand cDNA Synthesis Kit (Thermo Fisher Scientific, Waltham, MA, U.S.A.). Subsequently, with the cDNA as the template, quantitative real-time polymerase chain reaction (qRT-PCR) was performed using SYBR Premix ExTaq™ (TaKaRa, Otsu, Shiga, Japan). Relative gene expression was calculated by the 2^−ΔΔ*C*_t_^ method. GAPDH served as the internal reference. The primer sequences were as follows; GAPDH: 5′-AGAAGGCTGGGGCTCATTTG-3′ (forward), 5′-AGGGGCCATCCACAGTCTTC-3′ (reverse); CCNB2: 5′-CAACCCACCAAAACAACA-3′ (forward), 5′-AGAGCAAGGCATCAGAAA-3′ (reverse).

### Evaluation of the expression of CCNB2 in LGG

We verified the differential expression of CCNB2 between LGG samples and paracancerous samples in GEPIA2 (http://gepia2.cancer-pku.cn/) and TIMER (https://cistrome.shinyapps.io/timer/) databases. The GEPIA2 platform includes RNA sequencing data from 9736 tumor tissues and 8587 normal tissues from TCGA and GTEx databases. The main functions provided for evaluation included gene expression analysis, gene correlation analysis, survival analysis, similar gene prediction, and dimensionality reduction analysis. The mRNA expression of CCNB2 in LGG and various cancers was detected by the GEPIA2 and TIMER databases.

### Clinical correlation and prognostic analysis of CCNB2

The Chinese Glioma Genome Atlas (CGGA; http://cgga.org.cn/analyse/WEseq-data.jsp) database is a user-friendly web application for data storage and analysis of brain tumor datasets of more than 2000 samples from the Chinese population. The database includes full exome sequencing, DNA methylation, mRNA sequencing, mRNA microarray, microRNA (miRNA) microarray, and matched clinical data. The analysis tool allows users to browse DNA mutation profiles, mRNA/miRNA expression profiles and methylation profiles, and also allows correlation and survival analyses in specific glioma subtypes. To verify the relationship between CCNB2 and the prognosis of LGG, we explored the correlation of clinical variables and prognosis using CCNB2 and LGG data extracted from in the CGGA and GEPIA2 platforms.

### Univariate and multivariate Cox analyses

R (version 3.6.3) was used for all analyses. After downloading all data relative to LGG from TCGA, we excluded patients with incomplete clinical data, and used the ‘survival’ package in R to determine associations with clinical outcomes. Variables included gender; age; WHO grade; IDH mutational status; 1p/19q codeletion, and CCNB2.

### Evaluation of CCNB2 expression in cells and tissues

HPA, an open access database for mapping all human proteins in organs, tissues, and cells, by integrating various combinatorial techniques. The mRNA expression of CCNB2 in organs, tissues, and tumor samples was detected using a human protein map with data extracted from the TIMER database. The Broad Institute Cancer Cell Line Encyclopedia (CCLE) database is a tumor genomics research project led by the Broad Institute Research Institute, which collects the taxonomic data from more than 1000 tumor cell lines. We used both databases to verify the expression level of CCNB2 at different levels.

### Correlation analysis between CCNB2 and immune infiltration

Immune cell infiltration is closely associated with the prognosis of LGG. With the rise in targeted therapy, an in-depth study of the molecular mechanisms involved in the occurrence and development of LGG for the molecular targeted therapy of LGG has gradually emerged. TIMER applies a deconvolution method to infer the abundance of infiltration level from gene expression profiles [16]. We assessed the correlation of CCNB2 expression with the abundance of six types of immune cells (CD4+ T cells, CD8+ T cells, B cells, neutrophils, and macrophages) in LGG via the TIMER algorithm.

### Gene set enrichment analysis of CCNB2

To explore important pathways enriched between the high and low expression of CCNB2 in LGG, we carried out Gene Set Enrichment Analysis (GSEA). GSEA used ‘h.all.v.7.1.symbols.gmt’ as the reference gene set, and GSEA was carried out using GSEA 4.0.3, and 1000 genome replacements were carried out to achieve a standardized enrichment score. A nominal *P*-value <0.05 and a false discovery rate <0.05 were considered significant results for each analysis.

### Quantitative real-time PCR

Total RNA was extracted from tissues by TRIzol reagent (Invitrogen, Grand Island, NY, U.S.A.) and cDNA synthesis was further conducted according to the reverse transcription kit (Takara Bio, Shiga, Japan). Relative expression was detected using SYBR Green PCR Master Mix (Takara Bio, Shiga, Japan) on Bio-Rad CFX96GRT-PCR system. The calculation of relative quantification was performed by 2^−ΔΔ*C*_t_^ cycle threshold method. The primer sequences were as follows (‘F’ represents ‘forward’; ‘R’ represents ‘reverse’); GAPDH: 5′-AGAAGGCTGGGGCTCATTTG-3′ (F), 5′-AGGGGCCATCCACAGTCTTC-3′ (R); CCNB2: 5′-CAACCCACCAAAACAACA-3′ (F), 5′-AGAGCAAGGCATCAGAAA-3′ (R).

### Statistical analysis

All data were statistically analyzed by GraphPad Prism 4.0 (GraphPad Software Inc, U.S.A.) and R, which were presented as mean ± SD. Comparisons between groups were accomplished by Student’s *t* test. Comparisons between two or two groups using analysis of variance (ANOVA). Log rank test is used for survival analysis. For categorical variables, Chi-square tests were adopted for analysis. *P*<0.05 was considered statistically significant.

## Results

### CCNB2 expression in different human cancers

**Figure 1:**
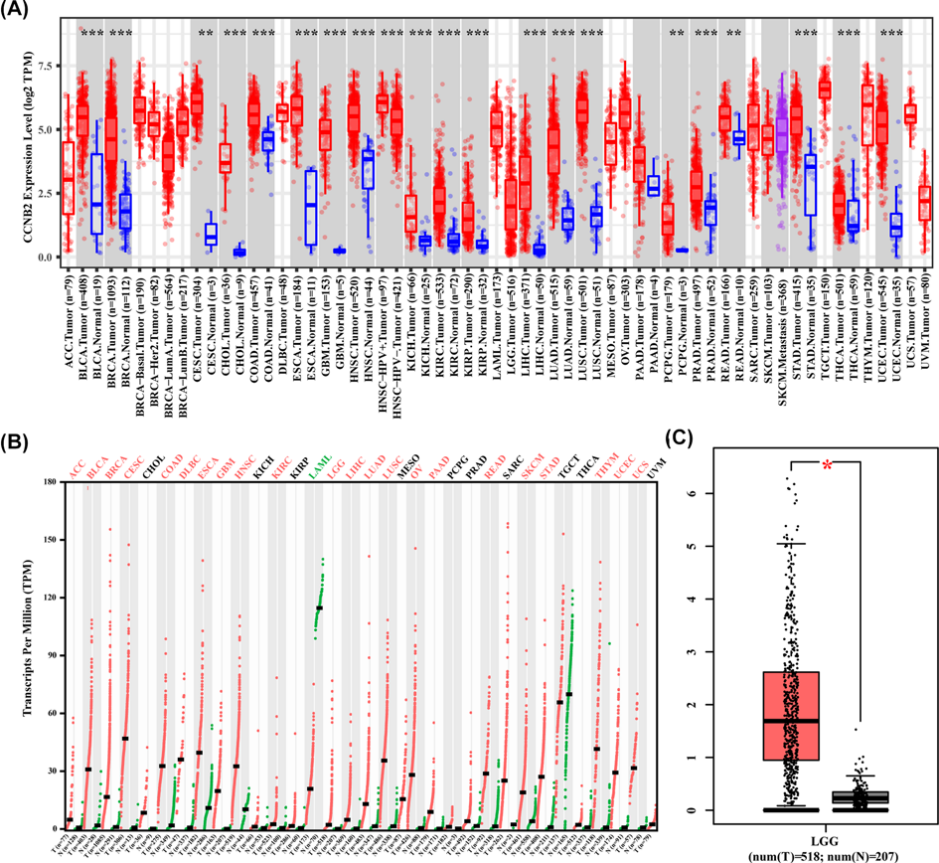
Differential expression of CCNB2 in various cancers (**A**) CCNB2 expression levels in various types of cancer from the TIMER database. (**B**) CCNB2 expression levels in various types of cancer from the GEPIA2 database. (**C**) Expression of CCNB2 is down-regulated in LGG from GEPIA2 database (*P<0.05; **P<0.01; ***P<0.001).

The results of CCNB2 and expression in various cancers showed that compared with the normal tissues, the expression of CCNB2 increased in BLCA, BRCA, OV, GBM, UCEC and many other cancers, but only decreased in LAML (Figure 1A). The results of GEPIA2 databases showed that the expression of CCNB2 in LGG was higher than that in normal tissues (Figure 1B,C).

### Clinical correlation and prognostic evaluation of CCNB2 and lower grade gliomas

**Figure 2:**
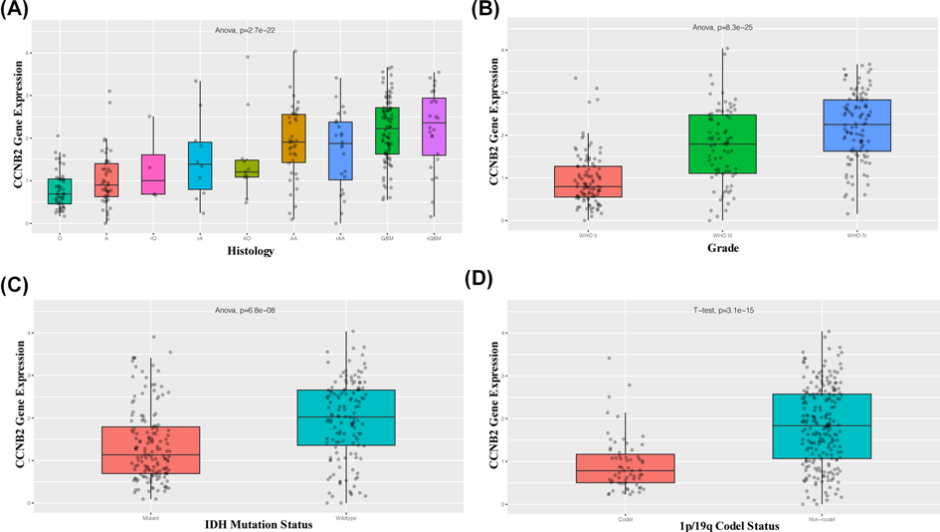
Correlation between CCNB2 mRNA expression and clinical indexes from mRNAseq_325 dataset CCNB2 mRNA expression is stratified by histology (**A**), WHO grade (**B**), IDH mutation status (**C**) and 1p/19q codel status (**D**).

**Figure 3:**
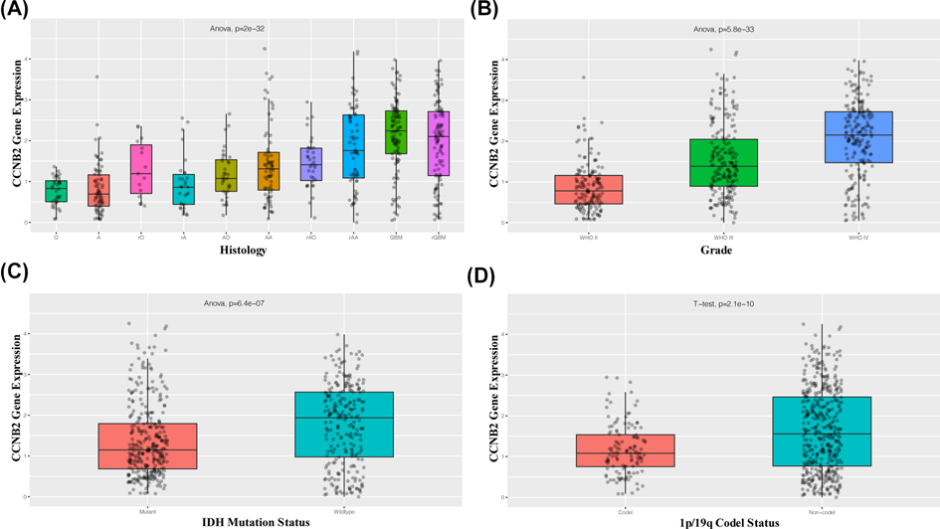
Correlation between CCNB2 mRNA expression and clinical indexes from mRNAseq_693 dataset CCNB2 mRNA expression is stratified by histology (**A**), WHO grade (**B**), IDH mutation status (**C**) and 1p/19q codel status (**D**).

**Figure 4:**
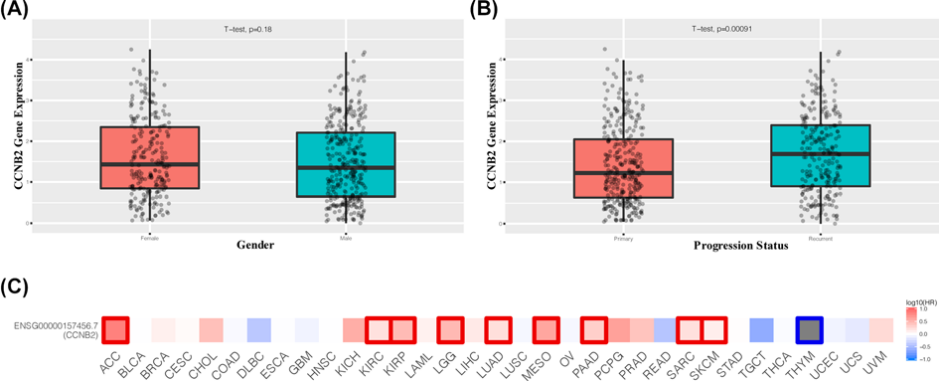
Association with CCNB2 mRNA expression and clinicopathologic characteristics (**A**) Correlation between CCNB2 mRNA expression and gender from CGCC database. (**B**) Correlation between CCNB2 mRNA expression and progression status from CGCC database. (**C**) The prognostic value of CCNB2 in major cancers. Red indicates that it has obvious prognostic value.

**Figure 5:**
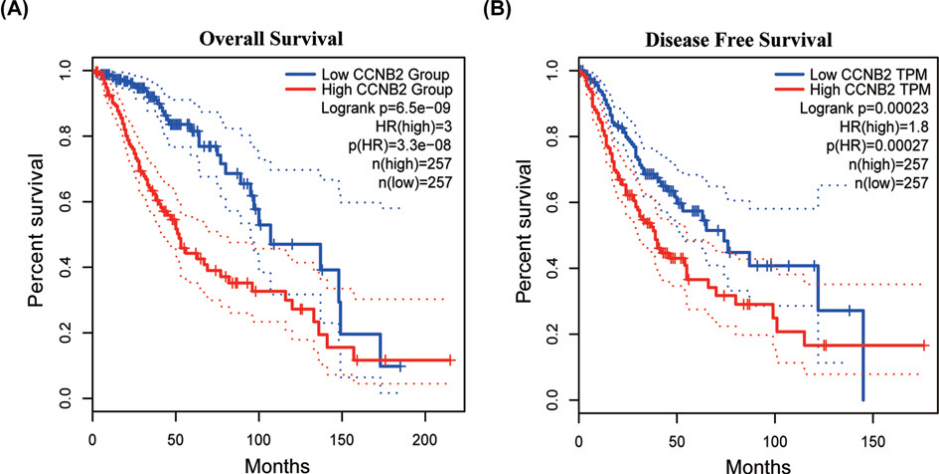
Survival analysis with CCNB2 mRNA expression (**A**) Survival curves of OS and (**B**) DFS comparing the high and low expression of CCNB2 in LGG in the GEPIA2.

The results of correlation analysis of clinical variables between CCNB2 and lower grade gliomas showed that in both the mRNAseq_325 dataset and mRNAseq_693 dataset, the expression of CCNB2 was correlated with different histological grades, age, IDH mutational status, and 1p/19q codeletion status (Figures 2 and 3). The expression of CCNB2 was not associated with sex (Figure 4A), but was closely related to the state of disease progression (Figure 4B). The results of the ‘Survival map’ in GEPIA2 revealed that CCNB2 was closely associated with the prognosis of ACC, GBM, LGG and PAAD (Figure 4C). The correlation analysis between CCNB2 and LGG prognosis showed that in the GEPIA2 datasets, higher CCNB2 levels were closely related with worse prognosis of LGG in overall survival (OS) and disease-free survival (DFS) (Figure 5A,B), which suggests that it may be a potential prognostic marker of LGG.

### CCNB2 was an independent prognostic factor for LGG in TCGA dataset

Univariate Cox results showed that age, WHO grade, IDH mutational status, 1p/19q codeletion and CCNB2 expression could be used as prognostic factors for LGG. Further multivariate Cox analysis showed that CCNB2 could be used as an independent prognostic factor for LGG in TCGA (*P*=0.007) (Table 1).

**Table 1:** Univariate and multivariate Cox regression analysis for CCNB2

Characteristics	Total (*n*)	Univariate analysis		Multivariate analysis	
		HR (95% CI)	*P*-value	HR (95% CI)	*P*-value
Gender (male vs. female)	527	1.124 (0.800–1.580)	0.499		
Age (>40 vs. ≤40)	527	2.889 (2.009–4.155)	**<0.001**	2.860 (1.869–4.377)	**<0.001**
WHO grade (G3 vs. G2)	466	3.059 (2.046–4.573)	**<0.001**	1.751 (1.107–2.771)	**0.017**
IDH status (Mut vs. WT)	524	0.186 (0.130–0.265)	**<0.001**	0.328 (0.208–0.519)	**<0.001**
1p/19q codeletion (non-codel vs. codel)	527	2.493 (1.590–3.910)	**<0.001**	1.722 (1.023–2.901)	**0.041**
CCNB2 (high vs. low)	527	2.995 (2.051–4.374)	**<0.001**	1.852 (1.180–2.908)	**0.007**

Abbreviations: CI, confidence interval; HR, hazard ratio. Bold values indicate *P*<0.05.

### Expression of CCNB2 in tissues and cells

**Figure 6:**
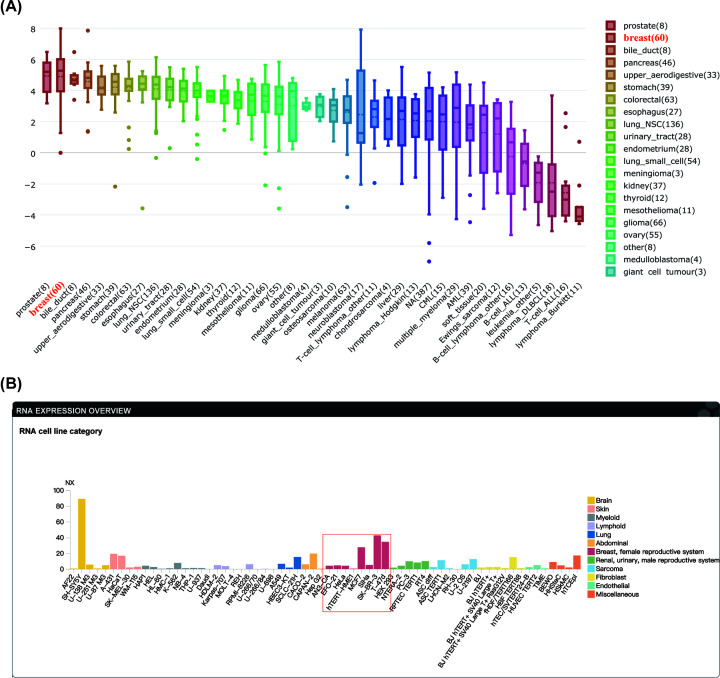
Validation of expression of CCNB2 in relevant human tissues from the RNA level and the protein level (**A**) CCNB2 mRNA expression in human tissues. (**B**) CCNB2 RNA expression in human cell lines.

**Figure 7:**
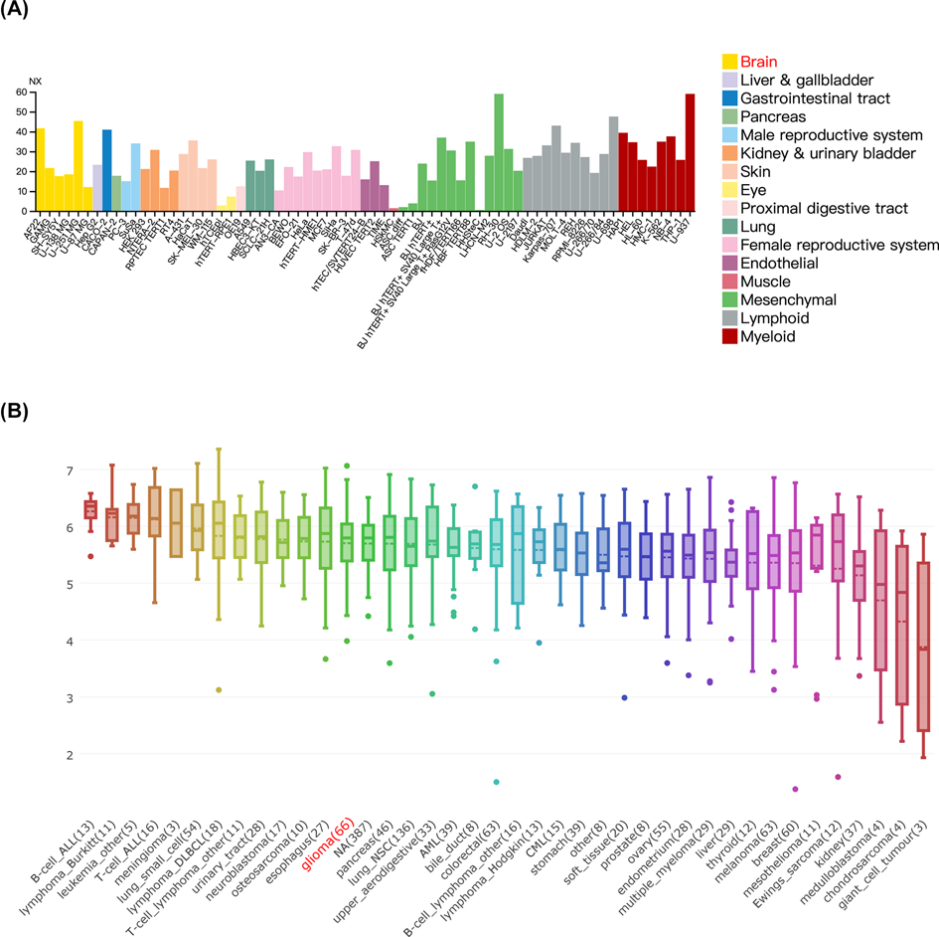
CCNB2 expression was significantly up-regulated in LGG tissues and cell lines (**A**) The expression of CCNB2 in different cell lines by HPA. (**B**) CCNB2 is highly expressed in glioma cell lines compared with other cancer types. Data were obtained through the CCLE.

The expression of CCNB2 was higher in male-sex specific tissues, blood and bone marrow, and lymphoid tissues, but was lower in female tissues, and was only expressed in the proximal digestive tract and in the gastrointestinal tract. The results of expression at the protein level in human normal tissues were similar to those at the mRNA level in males, and increased in endocrine tissues, the proximal digestive tract, and the gastrointestinal tract in females (Figure 6). The expression of CCNB2 varied across different cell lines. Compared with normal human epithelial nerve cells AF22, expression of CCNB2 was higher in U-251MG than in U-87MG. In the 66 cells related to glioma, we found that the average expression of CCNB2 was higher, and further, CCNB2 expression was higher than that observed in colorectal and stomach cancer cell lines, but was lower than the expression in esophagus cancer or in B-cell ALL cells (Figure 7).

### Correlation between CCNB2 expression and immune cell infiltration in LGG

**Figure 8:**
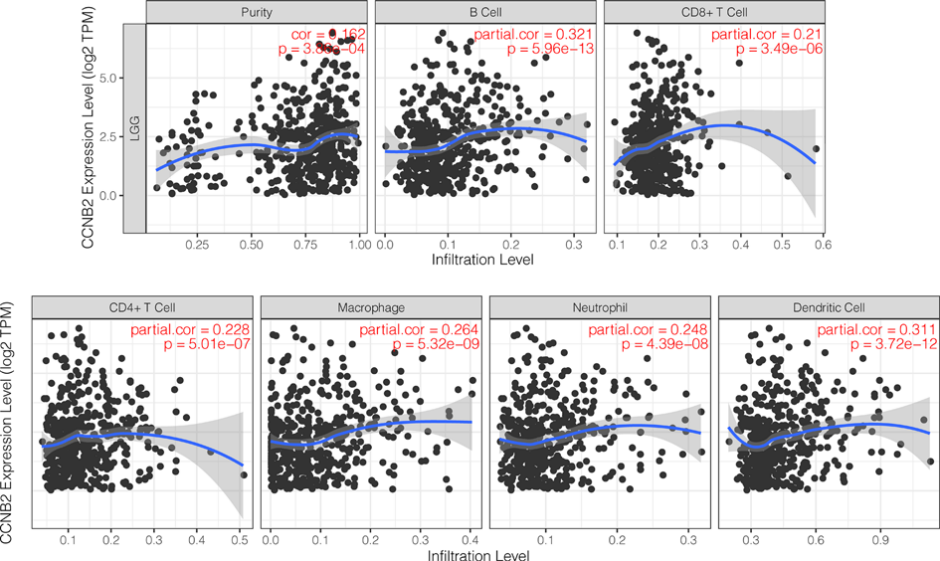
Correlation of CCNB2 expression with immune infiltration levels in LGG

**Figure 9:**
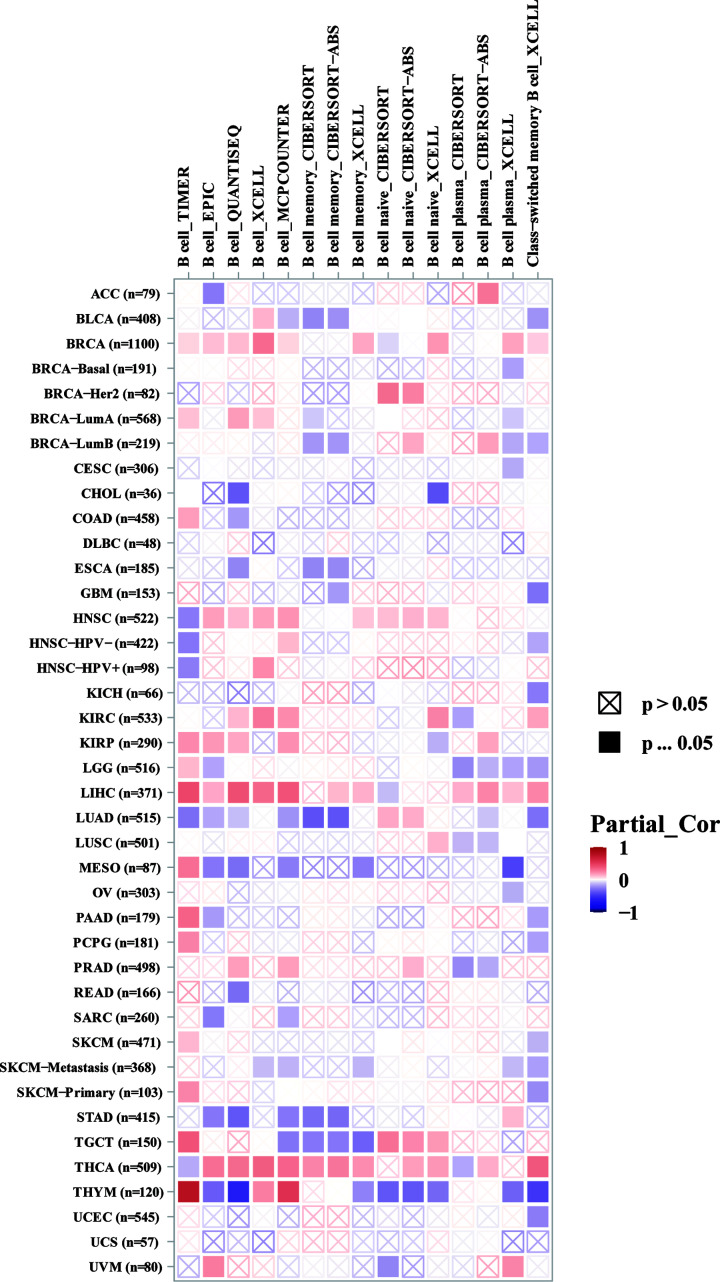
Correlation between CCNB2 and B-cell subtype

To investigate the relationship between CCNB2 and the diverse immune infiltrating cells, we focused on the correlations between CCNB2 and immune marker sets of different immune cells subsets present in LGG in the TIMER database. CCNB2 expression was significantly correlated with B cells (cor = 0.321, *P*=5.96e-13) (Figure 8), and more specifically, in terms correlation of LGG expression and B-cell subtypes, we found that there was a higher relationship between LGG and B cells, these findings indicated that clinical immunotherapy should play closer attention to effects on B cells (Figure 9).

### GSEA of CCNB2

**Figure 10:**
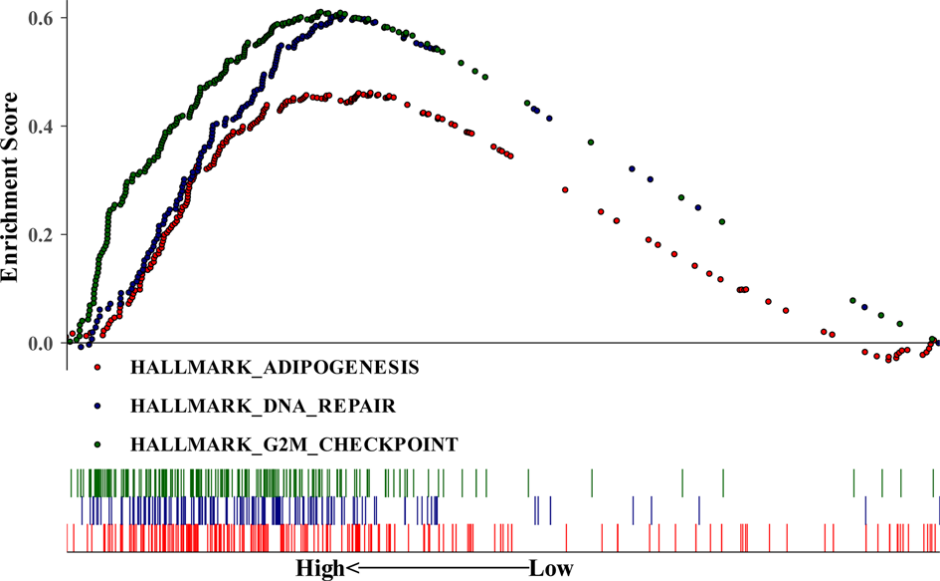
Enrichment plots from GSEA GSEA results showing differential enrichment of genes in KEGG with high CCNB2 expression.

Previous studies have confirmed that LGG patients with higher CCNB2 expression have a poorer prognosis; thus, we focused on the pathway enrichment analysis in LGG patient samples having higher expression of CCNB2. The results of single-gene GSEA showed that the higher expression of CCNB2 was mainly enriched in three pathways: adipogenesis, DNA repair and G2M_checkpoint (Figure 10).

### Validation of CCNB2 expression in independent LGG cohorts

**Figure 11:**
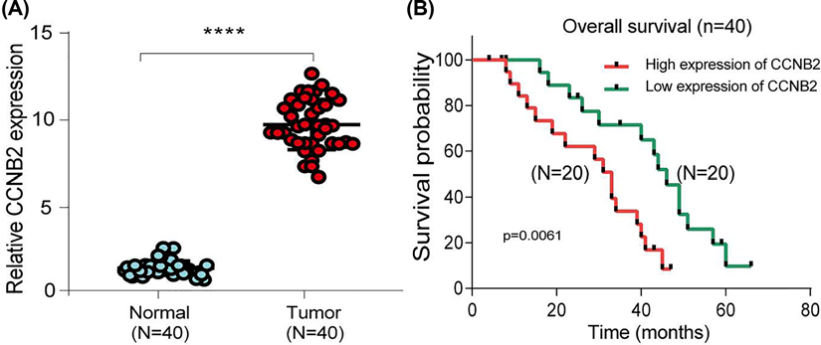
qRT-PCR showed that CCNB2 expression was up-regulated in LGG tissues and correlation with OS time (**A**) Expression of CCNB2 in 40 normal and tumor samples. (**B**) Kaplan–Meier OS analysis of Chinese LGG patients based on expression of CCNB2. ****P<0.0001.

We used qRT-PCR to characterize the expression of CCNB2 in LGG tissues and paracancerous tissues. CCNB2 expression in LGG tissues was markedly lower than in paracancerous tissues (Figure 11A). We then divided patients with LGG into high- and low-expression groups according to the median CCNB2 expression (20 patients with high expression and 20 patients with low expression) and explored the effects of CCNB2 on prognosis. Higher expression of CCNB2 was significantly associated with poorer OS (Figure 11B).

## Discussion

In our study, we used GEPIA2 and TIMER databases to examine the expression of CCNB2 in different cancers. The differential expression of CCNB2 in cancer and normal tissues is detected across a variety of cancers, a finding that was observed in both databases. We then verified the expression of CCNB2 in LGG tissue and in normal tissue samples by qRT-PCR. Our study showed that the expression of CCNB2 in LGG was higher than that in normal tissues.

Previous reports have described oncogenes related to the pathogenesis of gliomas [17,18]; however, few reports have evaluated the clinical and prognostic value of CCNB2 in LGG. The close relationship between the high expression of CCNB2 and the poor prognosis of liver cancer and prostate cancer stimulated our interest in its role in LGG [11,12]. Our results based on the analysis of datasets from the GEPIA2 web server revealed that LGG patients with low expression of CCNB2 had better OS and DFS than patients with high expression. Further, we also downloaded clinical data from TCGA database and explored whether CCNB2 could be used as a prognostic factor of LGG [19]. Both univariate and multivariate Cox analyses showed that CCNB2 was an independent prognostic factor in LGG. Our qRT-PCR results also confirmed that high expression of CCNB2 was associated with a worse prognosis than low expression of CCNB2. Overall, these findings indicated that CCNB2 is a potential and promising prognostic indicator for LGG.

Tumor infiltrating immune cells are an important part of the tumor immune microenvironment. Several studies have reported their effects on the biological behavior and survival rate of patients with LGG [20–22] and tumor-infiltrating immune cells have become a key regulator of the growth and progression of LGG [23,24]. In HCC, the expression of CCNB2 was shown to be positively correlated with the infiltration of CD4+ T cells and CD8 T cells [25]. In this study, we observed that the expression of CCNB2 was positively correlated with B-cell levels. B cells can differentiate into plasmablast-like cells in melanoma, which express T cells and recruit chemokines such as CCL5, and notably, CCL5 is the key factor involved in the survival of LGG stem cells [26], thus we speculated that it may also have a similar role in LGG. Although further studies are needed to verify our hypothesis, our results indicated that CCNB2 participates in the tumor immune microenvironment mainly by regulating B cells.

To further explore the pathogenesis of CCNB2 in LGG, we used GSEA to analyze the main enrichment pathways of high expression of CCNB2. The obesity epidemic has focused significant attention on adipose tissue and the development of fat cells, also known as adipogenesis [27]. Further, obesity and cancer are inextricably linked as there is evidence that obesity is associated with an increased risk of cancer in at least 13 anatomical sites [28]. Therefore, the intervention and treatment of obesity may contribute to a better prognosis of patients with LGG. Abnormalities in DNA repair can promote the occurrence and development of cancer. Similarly, these abnormalities are also considered as potential targets of cancer treatment [29]. As an enriched pathway in LGG patients with poor prognosis, it should receive greater attention. Cancer cells usually have defective G_1_-S checkpoints and rely on functional G_2_-M checkpoints for DNA repair [30]. Therefore, in the clinical treatment of LGG, we should specifically select G_2_-M checkpoint inhibitors, which may have a better therapeutic effect.

It is worth noting that our analysis has inevitable limitations. We only validated our findings in three databases, more specific verification may require a larger sample size. Further, we only verified the expression and prognosis of CCNB2 at the RNA level, the next step is to verify the expression and prognosis at the protein level. Our sample size was relatively small; thus, the results also have some limitations. Finally, although we preliminarily explored the biological process of CCNB2 in gliomas by GSEA, the detailed mechanism underlying the relationship between CCNB2 expression and LGG progression requires further study.

## Conclusion

CCNB2 is up-regulated in LGG, and the low expression of CCNB2 indicates a good prognosis in patients with LGG. In addition, the expression of CCNB2 may help regulate B cells, and plays an important role in tumor microenvironment. In summary, CCNB2 may be used as a promising biomarker for defining the prognosis of patients with LGG.

## Data Availability

All dataset used and/or analyzed during the current study are available from the corresponding author on reasonable request.

## Abbreviations

CCNB2: Cyclin B2
CGGA: Chinese Glioma Genome Atlas
DFS: disease-free survival
GBM: glioblastoma
GEPIA2: Gene Expression Profiling Interactive Analysis 2
GSEA: gene set enrichment analysis
GTEx: Genotype-Tissue Expression
HPA: Human Protein Atlas
LGG: low-grade glioma
miRNA: microRNA
OS: overall survival
qRT-PCR: quantitative real-time polymerase chain reaction
WHO: World Health Organization
